# Potential Application of Self-Assembled Peptides and Proteins in Breast Cancer and Cervical Cancer

**DOI:** 10.3390/ijms242317056

**Published:** 2023-12-02

**Authors:** Shidong Zhang, Meiqi Chen, Zijun Geng, Tianjia Liu, Shuangyang Li, Qixuan Yu, Lingling Cao, Da Liu

**Affiliations:** School of Pharmacy, Changchun University of Chinese Medicine, Changchun 130117, China; zhang_shidong@yeah.net (S.Z.); 15567958349@163.com (M.C.); jimmyg_98@163.com (Z.G.); 18744031073@163.com (T.L.); lisyang1206@163.com (S.L.); yuqx0221@163.com (Q.Y.)

**Keywords:** self-assembled peptides and proteins, breast cancer, cervical cancer, targeted cancer therapy, drug delivery, natural products

## Abstract

Ongoing research is gradually broadening the idea of cancer treatment, with attention being focused on nanoparticles to improve the stability, therapeutic efficacy, targeting, and other important metrics of conventional drugs and traditional drug delivery methods. Studies have demonstrated that drug delivery carriers based on biomaterials (e.g., protein nanoparticles and lipids) and inorganic materials (e.g., metal nanoparticles) have potential anticancer effects. Among these carriers, self-assembled proteins and peptides, which are highly biocompatible and easy to standardize and produce, are strong candidates for the preparation of anticancer drugs. Breast cancer (BC) and cervical cancer (CC) are two of the most common and deadly cancers in women. These cancers not only threaten lives globally but also put a heavy burden on the healthcare system. Despite advances in medical care, the incidence of these two cancers, particularly CC, which is almost entirely preventable, continues to rise, and the mortality rate remains steady. Therefore, there is still a need for in-depth research on these two cancers to develop more targeted, efficacious, and safe therapies. This paper reviews the types of self-assembling proteins and peptides (e.g., ferritin, albumin, and virus-like particles) and natural products (e.g., soy and paclitaxel) commonly used in the treatment of BC and CC and describes the types of drugs that can be delivered using self-assembling proteins and peptides as carriers (e.g., siRNAs, DNA, plasmids, and mRNAs). The mechanisms (including self-assembly) by which the natural products act on CC and BC are discussed. The mechanism of action of natural products on CC and BC and the mechanism of action of self-assembled proteins and peptides have many similarities (e.g., NF-KB and Wnt). Thus, natural products using self-assembled proteins and peptides as carriers show potential for the treatment of BC and CC.

## 1. Introduction

Recent medical developments include precision medicine and nanomedicine, which are also popular research subjects. Compared with traditional drugs and conventional drug delivery methods, nanoparticle-based drug delivery systems can improve drug stability and bioavailability and extend drug half-life. In tumor therapy in particular, nanocarriers reduce toxic side effects, improve biosafety and the ability to target tumors, and also provide conditions for precision medicine. However, premature drug leakage remains a challenge [1]. Various nanoparticle-based drug delivery vehicles, such as protein nanoparticles, lipids, and metal nanoparticles have been developed [2,3]. Among these vehicles, self-assembled peptides and proteins have exhibited attractive features in drug delivery, such as structural–functional versatility and biocompatibility [4]. Self-assembled proteins and peptides are biodegradable and have little toxicity compared to non-biological materials; when proteins and peptides hydrolyze into amino acids in the body, they are taken up and utilized by the surrounding tissues [5]. Numerous self-assembled peptides and proteins have been proven to have potential for cancer-targeted therapy. Because their therapeutic effect is superior to traditional treatment, they offer hope for the treatment of many cancers whose prognosis remains unsatisfactory.

For women worldwide, breast cancer (BC) has the highest incidence and mortality rate among all cancers, posing a threat to safety and life expectancy [6,7]. Because of advances in the clinical management of BC patients, the rising trend of mortality from BC has slowed down. However, the global incidence of BC is still increasing, and while the incidence is higher in developed countries, the mortality rate is higher in developing countries [8,9]. Risk factors for BC primarily include hormone levels, lifestyle, and oral contraceptive use [10]. Distant metastasis is the main cause of death in patients with BC. Once BC metastasizes, it becomes difficult to treat [11,12]. Cervical cancer (CC) is also one of the most common cancers in women, with the Global Cancer Statistics 2020 showing that CC is the most commonly diagnosed cancer in women in 23 of 185 countries—second only to BC (159 of 185)—and is the deadliest cancer in women in 36 countries [7]. Human papillomavirus (HPV) is a necessary cause of CC, and 12 types of HPV are classified as class-1 carcinogens by the International Agency for Research on Cancer, among which HPV-16 and HPV-18 have the highest carcinogenicity [13]. Other ancillary causes of CC include the long-term use of oral contraceptives, multiple births, a poor dietary structure, and an unhealthy lifestyle [14]. CC remains the leading cause of cancer deaths among women in some low- and middle-income countries, although with early intervention, CC is almost entirely preventable [15]. Therefore, the development of treatment modalities for BC and CC should not be neglected.

Given the recent advances in science and technology, the development of cancer therapeutics based on nanotechnology has become a focus of intense research. Self-assembled proteins and peptides have attracted increased attention because of their outstanding performance, and the research and development of cancer therapeutic platforms based on self-assembled proteins and peptides addresses both BC and CC. Natural products have great potential in the treatment of breast and cervical cancer, such as soy (genistein and soy sapogenins), pomegranate (ellagitannins), mangosteen (mangosteen glycosides), citrus fruits (naringenin), apples (2 alpha-hydroxyursolic acid), and cruciferous vegetables (isothiocyanates), among others. Interestingly, natural products loaded with self-assembled proteins produce a marvelous boost. In other words, natural products, when combined with self-assembling proteins, are not negligible for the therapeutic prospects of breast and cervical cancers.

## 2. Common Self-Assembled Proteins and Peptides in Cancer Therapy

**Figure 1:**
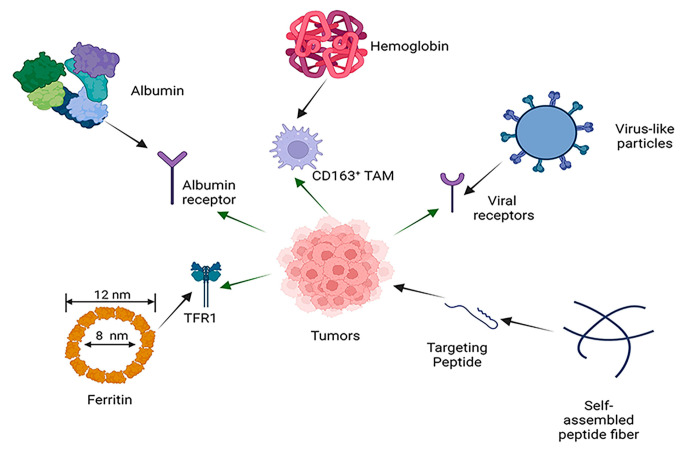
Common proteins and self-assembling peptides that act on cancer cells.

Peptide-derived self-assembled molecules have the potential to act as drug nanocarriers, and the different structures of these nanoparticles confer characteristics that could be advantageous in different contexts of nanomedicine. Multiple self-assembled proteins and peptides, such as albumin, ferritin, and virus-like particles (VLPs), have applications in cancer therapy (Figure 1) and are described below.

### 2.1. Albumin

Albumin is the most abundant protein in plasma, and several types of albumins have been isolated, including ovalbumin (OVA), human serum albumin (HSA), bovine serum albumin (BSA), and rat serum albumin (RSA) [16]. Because albumin is biodegradable, non-toxic, and immunogenic, and because its amino acid residues can be coupled to carry drugs, it has the potential to act as a nanodrug carrier. Accordingly, several albumin-based therapeutic regimens have been approved by the US Food and Drug Administration (FDA) for clinical treatment [17]. In addition, the expression levels of albumin receptors such as Gp18, Gp30, Gp60, and SPARC are abnormally elevated in cancer cells. Through an interaction between albumin receptors and albumin, albumin-based drug carriers are able to target tumors to a certain extent [18,19,20,21]. The preparation of hydrogels from albumin is another strategy for drug delivery. Recently, cross-linkable albumin-based hydrogels (CABH) have been prepared by synthesizing an N-hydroxysuccinimide (NHS)-activated linker, which can release piggyback drugs in a weakly acidic environment [22].

### 2.2. Ferritin

Ferritin consists of 24 subunits of heavy and light chains that can self-assemble into a highly symmetric nanocage with a particle size of 12 nm and an internal diameter of 8 nm [23]. The heavy and light chains of ferritin can form ferritin in any ratio, and this ratio is both species- and tissue-specific. Mediated by heavy-chain ferritin, ferritin binds to transferrin receptor 1 (TFR1) for cellular uptake [24]. TRFR1 is expressed at low levels in normal cells but has an abnormal overexpression state in common cancers, such as BC and lung cancers [25,26]. Hence, heavy-chain ferritin vehicles are tumor-selective without the addition of targeting ligands [27]. In addition, as a self-assembled protein, ferritin possesses various properties that render it suitable for use as a drug nanocarrier, including thermal stability (tolerance of high temperatures of 80–100 °C), pH stability (pH 3–10), monodispersity, and biodegradability [28]. In addition, artificial modification of ferritin can further improve the targeting of ferritin carriers, which substantially improves their safety characteristics [29,30]. Engineered ferritin nanocages can target cancer-associated fibroblasts in tumors, allowing for greater precision in cancer treatment [31].

### 2.3. Virus-Like Particles

In biotechnology research, VLPs are viral capsids that do not contain the complete viral genome and are incapable of infection or replication [32]. Because of their good immunological properties, VLPs have recently become useful tools for vaccine development, which has become one of their main applications [33]. Vaccines fabricated with the VLP of HPV have been licensed for clinical use for more than a decade, and to some extent, they have prevented HPV-related cancers like CC and oropharyngeal cancer [34,35,36]. In addition to preventive vaccines, VLPs have also been developed for preparing therapeutic vaccines for cancer [37,38]. VLPs can also be used for drug delivery, using their internal cavity for the treatment or imaging of tumors [39]. A VLP-based drug delivery system offers higher delivery efficiency and cancer inhibition than free drugs while ensuring biosafety [40]. Advances in bioengineering technology have allowed the synthesis of artificially designed VLPs with various functions to meet different needs [41].

### 2.4. Self-Assembled Peptides

Self-assembled peptides usually consist of short monomers or repetitive sequences of 8–16 amino acids that can spontaneously assemble into ordered nanostructures. As small molecules, common peptides are difficult to retain in the patient’s body for long periods when used to treat diseases [42]. However, after a series of artificial modifications, the self-assembled peptides can form structures that are more stable than traditional non-biological materials [43]. Drug-loaded pH-responsive hydrogels based on self-assembled peptides have been constructed for efficient drug delivery in the acidic microenvironment of tumors (pH 6.5) [44]. Researchers attached gold nanoparticles to self-assembled peptide assemblies to obtain biocomposites, using the targeting properties of self-assembled peptides and the light-triggered drug release properties of gold nanoparticles to achieve the dual targeting of BC cells [45].

These self-assembled peptides and proteins already possess certain targeting properties under natural conditions, and bioengineering can be used to introduce other modifications to these materials when designing them for production. This further enhances the targeting of nanocomplexes, giving them an advantage over other cancer therapeutics.

## 3. Classes of Drugs That Can Be Delivered by Self-Assembled Proteins and Peptides

**Figure 2:**
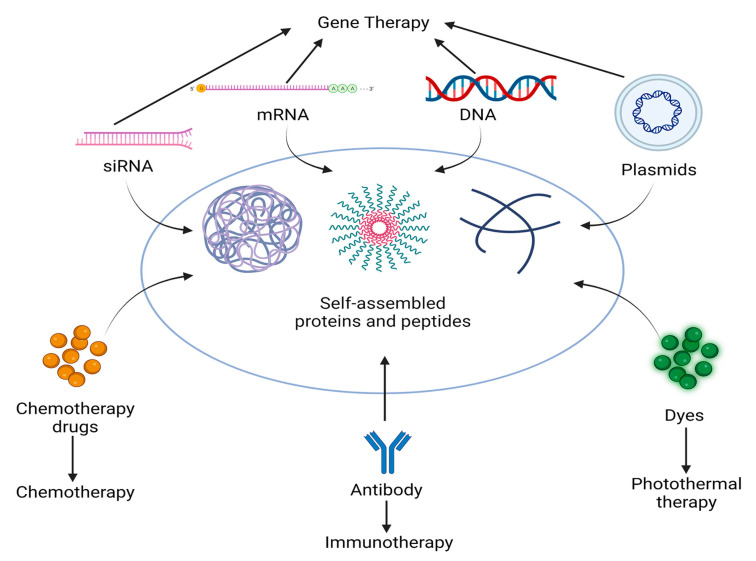
Targets and pathways of self-assembled peptides and protein carriers acting on diseases.

Self-assembled proteins and peptides can be modified for use as carriers to deliver a wide range of drugs, including conventional chemotherapy drugs, siRNA, mRNA, and DNA (Figure 2). Consequently, the use of self-assembled proteins and peptides as drug carriers has become nearly ubiquitous, being incorporated into cancer chemotherapy, gene therapy, and immunotherapy and appearing in the combinations of multiple therapeutic modalities. The discovery of these novel therapeutics has been a catalyst for the treatment of cancer and the development of protein self-assembly. It is believed that with the progress of research, including the discovery and application of new therapeutics, the treatment of cancer patients will be gradually improved.

Paclitaxel is one of the classic chemotherapy drugs. The development and market approval of Abraxane, a paclitaxel-albumin nanoparticle, was a milestone in nanopharmaceutical and albumin-based drug delivery technology [46]. Recently, Pt(II) thiosemicarbazone compound (C4) was optimized and loaded onto HSA for delivery to tumors, and in vivo experiments demonstrated that the protein complex inhibited tumor angiogenesis, expanding the potential of Pt drugs [47]. In addition, Joana B Ferrado et al. used BSA as a carrier for chrysin, a flavonoid compound that has been proven to have anticancer activity and characterized the obtained particles, which indicates that self-assembled proteins and peptides can also be used for natural product delivery [48].

The current research has identified an increasing number of aberrantly expressed genes that are inextricably linked to the development of cancer, and targeting these genes to reverse their abnormal expression status has also been shown to have an inhibitory effect on cancer. Liuwan Zhao et al. used cationic BSA as a carrier for the siRNA of S100A4 and further packaged it using exosomal membranes from autologous BC cells to increase the targeting ability and delivery efficiency [49]. As a result, the nanoparticles inhibited the expression level of S100A4 in lung tissues and exerted an inhibitory effect on postoperative BC metastasis in mice models. Another study delivered DNA using a Hedera arginine peptide (R11) to treat bladder cancer, increasing the stability and target specificity of DNA and achieving intra-bladder gene therapy [50]. In addition, an experiment in human bronchial epithelial-like cells demonstrated that self-assembled peptide complexes can transiently overexpress genes by delivering mRNA, which provides for the short-term recovery of some genes that are under-expressed in cancer [51].

However, siRNA, DNA, and mRNA have only a short-term therapeutic effect. The delivery of plasmids is one of the properties required for self-assembled proteins to be able to perform long-term gene therapy. Recently, Gil Jeong et al. prepared fusion peptides using nuclear localization signals and cell-penetrating peptides with calcium phosphate to transfect cells with large sequence plasmids containing Cas9 with higher transfection efficiency than Lipofectamine^®^ 2000 [52]. Experiments in mice showed that protein expression after delivery of plasmids using fusion peptides remained stable for a longer time than with liposome transfection. In another study, a self-assembled nanocarrier prepared with protamine and calcium carbonate was used to deliver CRISPR/Cas9 plasmids to tumors and achieve the knockdown of target genes in tumor cells [53].

Antibodies are potentially targeted drugs that can be used for cancers such as BC and ovarian cancer [54]. However, as stand-alone drugs for cancer treatment, antibodies have many shortcomings, such as a limited tumor-penetration ability and a short circulating half-life [55]. Nevertheless, the use of self-assembled proteins to deliver antibodies further enhances their targeting ability [56]. Previously, a self-assembled protein hexameric barrel was constructed to deliver antibodies to human CC cells, achieving their delivery to the cytoplasm [57].

In addition, researchers assembled HAS with D-α-tocopherol succinate and indocyanine green (ICG) into nanoparticles through interactions and used ion-pairing to carry doxorubicin (DOX) to construct a combined chemotherapeutic drug to achieve the targeted clearance of BC tumors in mice, without substantial toxicity [58].

Self-assembled peptides and proteins can carry different kinds of drugs, and when conditions allow, one complex can carry multiple drugs, making it possible to build multifunctional nanomedicines.

In the same way that ferritin, albumin, and virus-like particles are effective against BC and CC, many natural downstream products are also effective against these cancers through different mechanisms of action.

Natural products have been screened as anticancer agents because of their cost-effectiveness, low toxicity, and fewer side effects, and they are considered alternative treatment options for BC. Natural products show anticancer activity against BC by inhibiting angiogenesis, cell migration, proliferation, and tumor growth [59]. The cell cycle is blocked by the induction of apoptosis and cell death, downstream regulation of signaling pathways (e.g., Notch, NF-κB, PI3K/Akt/mTOR, MAPK/ERK, and NFAT-MDM2), and the modulation of the EMT process [60]. Natural products also act synergistically to overcome the problem of drug resistance, thus increasing their efficacy for BC therapy [61].

Laura reported that the design of Cur nanoparticles synthesized by alkaline incorporation inside a self-assembling variant (HFn) biopolymeric nanocage might yield soluble Cur endowed with high targeting efficiency toward cancer cells. The co-delivery of doxorubicin and the pH-sensitive curcumin prodrug by transferrin-targeted nanoparticles for BC treatment was reported [62]. Using a breast cancer xenograft mouse model, Tongxing Cui demonstrated that this co-encapsulation approach resulted in an efficient tumor-targeted drug delivery, decreased cytotoxic effects, and exhibited a stronger antitumor effect [63]. Luis Alberto Castillo-Díaz et al. demonstrated that self-assembling peptide hydrogels (SAPEs) were able to form nanostructures with a diameter of 20–200 nm. The SAPEs were adjuvanted with CpG-induced and expanded antigen-specific CD8+ T cells in mice [64]. Wei Zhang discovered a novel drug nanocarrier that reduced the survival of prostate cancer cells in vitro by slowly delivering DTX (which is highly concentrated) with GRP ligands on its surface [65]. Doxorubicin-loaded HFn acts as a “Trojan Horse”: HFn is internalized in cancer cells faster and more efficiently compared to free doxorubicin, then promptly translocated into the nucleus following the DNA damage caused by the partial release in the cytoplasm of encapsulated doxorubicin [66]. This self-triggered translocation mechanism allows the drug to be directly released into the nuclear compartment, where it exerts its toxic action. This approach is reliable and straightforward, providing an antiproliferative effect with high reproducibility [67]. Kai Xiao encapsulated hydrophobic drugs, such as PTX, and self-assembled them to form stable micellar nanoparticles [68].

Omega-3 fatty acids naturally occurring in fish, phytoresveratrol (3,5,40-trihydroxystilbene), epigallocatechin gallate (a polyphenolic compound found in green tea), and polyphenolic compounds from medicinal plants (*Oldenlandia diffusa*) and fruits (*Ziziphus jujuba*) show the potential for use in BC treatment [69]. Soybean (genistein and soy glycosides), pomegranate (ellagitannin), mangosteen (mangosteen glycosides), citrus fruits (naringenin), apples (2α-hydroxyursolic acid), and cruciferous vegetables (isothiocyanates) show potential for use in treating and preventing BC. Their activities involve several mechanisms, such as inhibiting the proliferation, migration, metastasis, and angiogenesis of tumor cells, inducing apoptosis and cell-cycle blockage, and sensitizing the tumor cells to radiotherapy and chemotherapy [70]. Nimbolide disrupts the interaction of RNF114 with one of its endogenous substrates, p21, the level of which is rapidly stabilized in BC cells in a p53-independent manner. Nimbolide can functionally access the E3 ligase protein–protein interaction site for potential cancer therapeutic effects and targeted protein degradation applications, particularly in the intrinsically disordered protein region [71]. Curcumin exerts anti-BC effects through a complex molecular signaling network involving the proliferation, estrogen receptor (ER), and human epidermal growth factor receptor 2 (HER2) pathways. The experimental evidence suggests that curcumin also regulates apoptosis and cell phase-related genes and microRNAs in BC cells [72].

In addition to biomarkers and therapeutic targets, miRNAs have been produced as novel drugs for the treatment of human diseases and the development of neoadjuvant therapies. Zhipin Liang and Yaguang Xi reported that TGF-β mediates the inhibitory signaling of SSA, a novel non-COX-inhibitory derivative of SS, which can be used to regulate the oncogenic miR-21 in cancers. ssA can reduce the phosphorylation of smad2/3, thereby blocking TGF-β signaling into the nucleus, leading to the downregulation of miR-21 transcription and inhibiting BC cell proliferation. Furthermore, combining proteins and natural products could be a new approach to the treatment of BC [73]. Ahmed et al. reported that natural products act as selective inhibitors of cancer cells by targeting oncogenes or altering miRNA expression. EGCG from tea was found to inhibit proliferative activity by regulating miR-16 and miR-21. Similarly, DIM found in Cruciferae was found to downregulate miR-92a, which regulates NFkB and prevents BC development. Plant-derived glycerol glycosides were found to upregulate miR-181c and miR-181d, which play a role in BC inhibition. These compounds also regulate miR-22, 29b and c, miR-30d, and 34a and 195. Quercetin (a flavonoid with anticancer activity) induces apoptosis in BC cells by regulating miR-16, 26b, 34a, let-7g, 125a, and miR-605, among others [74]. In addition, compounds such as radish sulfide, curcumin, genistein, resveratrol, lycopene, and epigallocatechin-3-gallate have been shown to promote cell cycle arrest and apoptosis in TNBC cells. They also inhibit epithelial–mesenchymal transition (EMT), which plays an important role in metastasis. Moreover, these natural compounds inhibit crucial pathways for cancer stem cells (CSC, a subpopulation with stem cell characteristics and a tumor-initiating propensity), such as NF-κB, PI3K/Akt/mTOR, Notch 1, Wnt/β-catenin, and YAP [75].

However, the clinical translation of natural products is often hampered by their poor stability, water solubility, and bioavailability. Attempts have been made to overcome these limitations, particularly through the use of nano-based drug delivery systems (NDDS), which are a new approach to the clinical application of natural products [76]. Natural products can achieve anti-CC effects through various mechanisms, including the inhibition of tumor cell proliferation, induction of apoptosis, inhibition of angiogenesis and telomerase activity, enhancement of immunity, and reversal of multidrug resistance [77]. Flavonoids, alkaloids, polyphenols, terpenoids, and quinones can act as anticancer agents. Natural products exert anti-CC functions mainly by inducing protective autophagy. Natural product modulation of autophagy in CC has notable advantages in inducing apoptosis, inhibiting proliferation, and reducing drug resistance in CC cells [78]. Given the limited options available to patients with recurrent CC, natural products are likely to be the therapeutic agents of choice for first-line metastatic disease or even as sensitizers for primary therapy to prevent recurrence. Optimal combinations and regimens need to be refined and balanced against the potential toxicity of these agents [59]. For instance, β-elemene inhibited cell proliferation and invasion and induced apoptosis via attenuation of the Wnt/β-catenin signaling pathway in CC cells. Copper oxide nanoparticles synthesized using *Azadirachta indica*, *Hibiscus rosa-sinensis*, *Murraya koenigii*, *Moringa oleifera*, and *Tamarindus indica* induced apoptosis and inhibited oxidation in HeLa cells at doses of 2 μg/mL, 5 μg/mL, 10 μg/mL, 25 μg/mL, 50 μg/mL, and 100 μg/mL for 48 h. The expression of p53, cytochrome c, caspase-3, -7, and -9, and PARP increased, whereas Bcl-2 and NF-κB decreased in HeLa cells by treating them with curcumin-loaded TPGS/F127/P123 mixed polymeric micelles isolated from Curcuma longa at a dose of 2 μg/mL for 48 h [79]. This treatment induced apoptosis at a dose of 50 μg/mL for 48 h. In addition, it inhibited cell metastasis and proliferation in CC cells by reducing VEGF, CDK2, and ERK1/2. Praeruptorin-B isolated from Peucedanum praeruptorum Dunn. downregulated NF-κB and MMP-2 and -9 in HeLa and SiHa cells [80]. The inhibition of cell metastasis was observed following the treatment with praeruptorin-B at doses of 40 μg and 60 μg over 24 h. Moreover, this treatment blocked Akt phosphorylation without affecting the MAPK pathway. These results suggest that praeruptorin-B could be an effective anticancer agent, particularly for CC. Park reported 64 natural products that exhibited apoptotic, anti-angiogenic, and anti-metastatic effects and were able to modulate multidrug resistance and miRNAs. Most natural products (47), including curcumin, rhodopsin, and penicillin, induced apoptosis. Four natural products, including xylin and xanthin, exhibited anti-angiogenic properties in CC cells. Five natural products, including EGCG, exhibited inhibitory effects on cancer metastasis. More natural product therapeutic mechanisms are presented in Table 1.

All of these indicate that natural products have good prospects and research significance in issues such as therapeutic prophylaxis and drug resistance in the treatment of CC.

## 4. Potential Applications of Self-Assembled Proteins and Peptides in BC and CC

Antitumor drugs can be divided into cytotoxic drugs, hormonal drugs, biological response modifiers, and so on. Cytotoxic drugs mainly kill tumor cells by inhibiting their growth and division. Representative drugs include cyclophosphamide, 5-fluorouracil, mitomycin, and so on. Hormonal drugs inhibit the growth and reproduction of tumor cells by regulating the level of hormones in the body. Representative drugs include tamoxifen, flutamide, treprostinil, etc. Biological response regulators can enhance the immune function of the body and regulate the immune response to achieve the purpose of tumor treatment. Representative drugs include interferon, in-terleukin-2, tumor necrosis factor, etc. Currently, the following drugs are used in the clinical treatment of breast cancer.

Cytotoxic drugs: cyclophosphamide, doxorubicin, docetaxel, methotrexate, and fluorouracil. Hormonal agents: ER-antagonists. tamoxifen, raloxifen, fulvestrant, elac-estrant, etc. Biological response modifiers such as the PI3K inhibitor capivasertib and the CDK4/6 inhibitor abemaciclib. 

Aromatase inhibitors are used in the management and treatment of BC. These drugs inhibit aromatase, a member of the cytochrome P450 superfamily of monooxygenases that catalyze the demethylation of androgenic carbon 19 to produce phenolic 18-carbon estrogens. In postmenopausal women, the conversion of androgens to estrogens via pathways in the adrenal glands, skin, muscle, and adipose tissue is the major source of estrogens. Aromatase inhibitors block this pathway, thereby suppressing estrogen levels in these women. BC cells also exhibit aromatase activity, a possible source of local estrogen in tumor cells. The inhibition or inactivation of aromatase suppresses serum estrogen levels, thereby reducing estrogen-mediated cancer cell proliferation in hormone receptor-positive BC [96]. Raloxifene is a drug used to treat postmenopausal osteoporosis and lower the risk of invasive BC in postmenopausal women [97]. Cyclophosphamide is a drug used primarily for the management and treatment of tumors, including multiple myeloma, sarcoma, and BC [98]. Pamidronate is an agent used in the management and treatment of moderate or severe hypercalcemia of malignant tumors, moderate-to-severe Paget’s disease of the bone, osteolytic bone metastases from BC, and osteolytic lesions from multiple myeloma [99]. Trastuzumab is a biologic antitumor drug that was one of the first available targeted chemotherapeutic agents when it was approved by the FDA in 1998 [100]. Tamoxifen is a selective estrogen receptor modulator (SERM) drug used to treat male and female BC and as a preventive drug for female BC [101]. Fulvestrant is a selective estrogen receptor degrader used in the management and treatment of advanced BC [102]. 

Adriamycin has strong antitumor effects, because its structure contains both fat-soluble anthracycline ligands and water-soluble flexible glycosaminoglycosides [103], acidic phenolic hydroxyl groups, and basic amino group. Its mechanism of action is that it can act directly on DNA, insert into the double helix of DNA, cause the latter to unravel, change the nature of the template of DNA, inhibit DNA polymerase, and thus inhibit both DNA and RNA synthesis [104,105]. Docetaxel has the same action as paclitaxel (PTX), which is an M-phase cycle-specific drug, where it promotes the polymerization of tubules into stable microtubules and inhibits their depolymerization, which significantly reduces the number of tubules and destroys the microtubule meshwork [106,107]. In vitro experiments have shown that it has cytotoxic effects on a variety of mouse and human tumor cell lines [108], with a broader antitumor spectrum than PTX, and can attenuate the expression of Bcl-2 and Bcl-xL genes [109]. Methotrexate, an organic compound with the chemical formula C20H22N8O5, is mainly used as an antifolate antitumor drug [110,111], which inhibits the growth and reproduction of tumor cells by hindering the synthesis of tumor cells through the inhibition of dihydrofolate reductase [112]. Fluorouracil, also known as 5-fluorouracil, with the chemical formula of C4H3FN2O2, is a pyrimidine analog, which belongs to a kind of antimetabolite drugs and is mainly used for the treatment of tumors [109,113]. Elacestrant is an estrogen receptor antagonist that binds to estrogen receptor-alpha (ERα). In ER-positive (ER+) HER2-negative (HER2-) breast cancer cells [114], elacestrant inhibits 17β-estradiol-mediated cell proliferation at concentrations that induce ERα protein degradation mediated through the proteasome pathway [115]. Elacestrant has demonstrated antitumor activity in vitro and in vivo, including in an ER+HER2- breast cancer model against fulvestrant and cell cycle protein-dependent kinase 4/6 inhibitors as well as in resistance models with mutations in the estrogen receptor 1 gene (ESR1) [116]. Truqap (capivasertib) is an oral selective adenosine triphosphate (ATP) competitive inhibitor of all three isoforms of the serine/threonine kinase AKT (AKT 1/2/3), which is designed to work by targeting mutations in the AKT1 gene, which is responsible for tumor growth and proliferation [117,118]. Abemaciclib, sold under the trade name Verzenio, is an inhibitor of CDK4/CDK6 [119], the kinases that bind to cell cycle proteins (Cyclins). In female receptor (ER)-positive breast cancer cell lines, Cyclin D1 and CDK4/6 promote retinoblastoma protein (Rb) phosphorylation, cell cycle progression and cell proliferation [120]. In vitro experiments demonstrated that sustained exposure to abecyclidine inhibited the phosphorylation of RB and prevented the cell cycle from G1 to S phase, leading to senescence and apoptosis [121,122].

The world’s first marketed antibody-coupled drug targeting the Trop-2 receptor, Topavir® (gosatuzumab), is a cell surface antigen that is highly expressed in many types of tumors, including more than 90% of breast and bladder cancers. The monoclonal antibody targeting Trop-2 is linked to the payload topoisomerase I inhibitor SN-38 by a hydrolyzable linker [123]. This unique design ensures potent activity in Trop-2-expressing cells and the adjacent microenvironment. As a next-generation ADC drug, gossatuzumab has an optimized structure and a number of mechanistic advantages: the payload, SN-38, is a DNA inhibitor with a potent killing effect; the high drug/antibody ratio of 7.6 allows for the delivery of a higher payload with good systemic tolerance; and the linker is a shorter, polyethylene glycol unit structure that improves water solubility of the drug, aids in the overcoming of drug resistance, and allows for the slow release of SN-38, enhancing drug resistance. SN-38 enhances drug tolerance and efficacy; the cleavable linker allows the drug to exert a bystander effect, which is also effective in killing Trop-2 negative tumor cells [123]. The indications, contraindications, and adverse effects of these agents are listed in Table 2

The discovery of self-assembled proteins and peptides means that drugs can be delivered accurately to the corresponding targets. Our previous article also introduced various natural products for the treatment of BC and CC, although they may not be able to achieve a rapid onset of action compared with synthetic drugs. Nevertheless, natural products do not tend to have serious side effects, and it would be interesting to combine natural products with self-assembled proteins and peptides.

**Figure 3:**
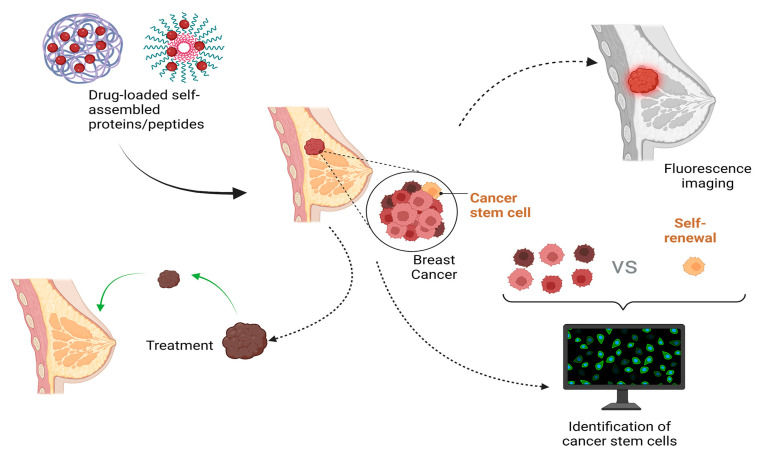
Self-assembled peptides and protein carriers for the treatment and diagnosis of BC.

At present, considerable research has focused on cancer therapeutics based on self-assembled proteins and peptides, with the two most common female cancers (BC and CC) being key research and development targets. In particular, numerous studies have shown the potential of self-assembled peptides and proteins for the treatment and diagnosis of BC, the deadliest cancer (Figure 3).

Surgical excision is the preferred treatment option for early-stage BC, yet nearly a quarter of patients require further surgery to reduce the risk of recurrence [124]. Therefore, a recent study reported the synthesis of a multifunctional nanoprobe, gadoiniumdiethylenetriaminepentaacetic acid/human serum albumin@ICG-bevacizumab, which is based on human serum albumin, to enhance the targeting effect of the nanoprobe by targeting the vascular endothelial growth factor A (VEGF-A) with bevacizumab [125]. This nanoprobe can be used not only for fluorescence imaging of tumor tissue to guide surgery but also to increase the sensitivity and targetability of radiation therapy.

TFR1 is highly expressed in several cancers, including BC and CC, which provide appropriate conditions for ferritin to be used as a targeted nanodrug carrier [126,127]. A recent study reported the use of ferritin cages to mount the flavonoid curcumin, which improved the stability and delivery efficiency of the bioactive and increased its oncogenic effect on BC in vivo and in vitro [128]. The hyperthermophilic halophile Archaeoglobus fulgidus can produce atypical ferritin, which is characterized primarily by a unique tetrahedral structure that results in four pore sizes—all close to 4.5 nm—and by its dissociation at low salt (NaCl) concentrations [129]. Based on these properties, self-assembled porous exoshells (tES) were prepared and used for bio-orthogonal catalysis of BC with horseradish peroxidase (HRP), which exploited its ability to encapsulate proteins and protect them from hydrolysis [130].

BC stem cells dominate BC’s development, drug resistance, and metastasis [131]. An analysis of BC stem cell counts can help predict the presence of metastatic trends in BC and provide guidance for its treatment and prognosis. In designing a scheme based on the advantages of self-assembled peptides, Yingying Tang et al. first captured BC cells using nucleolin adaptor AS1411, which was immobilized on the electrode surface, which subsequently bound self-assembled peptide nanofibers targeting the stemness marker CD44 to BC stem cells, and finally recruited electroactive silver nanoparticles to generate electrochemical signals [132]. This method of identifying BC stem cells was experimentally validated and demonstrated high sensitivity and specificity and thus may provide assistance in the clinical diagnosis of BC.

Triple-negative breast cancer (TNBC) comprises a range of BCs with different characteristics that lack an estrogen receptor, a progesterone receptor, and the human epidermal growth factor receptor 2 (ERBB2; formerly known as HER2). More aggressive and more likely to recur, TNBC accounts for approximately 20% of newly diagnosed BCs [133,134]. Due to the lack of hormone receptors and ERBB2, fewer treatment options are available for TNBC compared to other BC subtypes [135]. The BACH1 gene is highly expressed in the tumors of TNBC patients and is associated with mitochondrial metabolism, whereas heme has been previously shown to cause specific degradation of BACH1 and the consequent inhibition of TNBC [136,137]. Hence, Xuan Yang et al. sought to develop targeted therapeutic agents for the treatment of TNBC by combining heme and berberine derivatives, which are inhibitors of mitochondrial function, into self-assembled nanoparticles [138]. These authors utilized chondroitin sulfate to encapsulate the nanoparticles while relying on the affinity of chondroitin sulfate for CD44 to increase the targeting effect of the nanoparticles on TNBC [139]. The obtained self-assembled nanoparticles exhibited inhibitory effects on the proliferation, migration, and invasive ability of BC cells. Furthermore, they promoted the apoptosis of BC cells, which was caused by inducing mitochondrial damage. In vivo experiments revealed that the nanoparticles inhibited tumor growth and were more biosafe for administration than free berberine derivatives.

The epidermal growth factor receptor (EGFR) is another promising therapeutic target for TNBC. Earlier, a phage was found to produce the EGFR-targeting peptide GE11, which makes it feasible to use GE11 for targeting TNBC [140,141]. TNBC is the most metastatic type of BC, and bone metastasis is a common outcome [142]. Because of the hydrophobicity and low stability of anticancer drugs, the treatment of bone metastases derived from TNBC is challenging [143]. Therefore, there is a need to investigate hydrophobic drug delivery vehicles that can help increase the therapeutic effect of hydrophobic anticancer drugs. A breakthrough was achieved when a study reported the design of an amphiphilic self-assembled nanoplatform using stearic acid-modified GE11, which was named GENP [144]. In that study, GENP@DOX was constructed using GENP loaded with DOX, resulting in satisfactory loading efficiency, tumor cell targeting, cellular uptake, and sustained drug release. GENP@DOX exhibited a favorable antitumor effect in TNBC orthotopic tumors together with a higher biosafety profile and also substantially decreased the tumor size in mice models of TNBC bone metastasis. Recent research also found that GENP as a vector can independently inhibit the proliferation of BC cells through the PI3K/AKT signaling pathway downstream of EGFR, and these phenomena together demonstrate that GENP is an effective targeting therapeutic strategy for TNBC.

**Figure 4:**
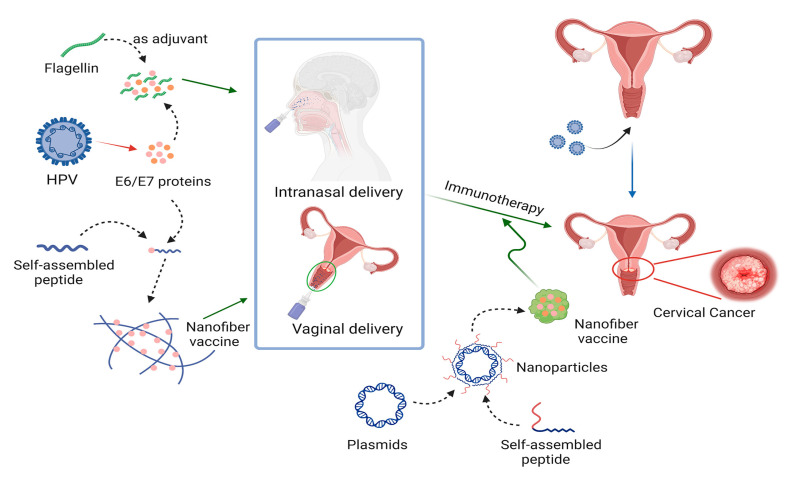
Preparation of a CC tumor vaccine using self-assembled peptides.

Immunotherapy is a widely used biological therapy in tumor treatment, as tumor vaccines can use the patient’s immune system to destroy cancerous cells by stimulating immune cells to recognize tumor-specific antigens [145]. Because of HPV’s inseparable relationship with CC, the primary method of targeted therapy for CC using self-assembled peptides involves tumor vaccines [146] (Figure 4).

The self-assembled peptide Q11 is not immunogenic and can be used for the intranasal delivery of antigenic epitopes, eliciting a greater CD8+ T cell response than subcutaneous immunization [147]. Thus, Q11 could be used to develop cancer vaccines. Recent research fused the oncogenic protein E744-62 of HPV-16 with the self-assembled peptide Q11 to prepare an E7-Q11 tumor vaccine for HPV-associated tumors [148]. In that study, CC orthotopic mice models were used as subjects, and E7-Q11 was administered by the intravaginal, intranasal, or subcutaneous routes. The results demonstrated that E7-Q11 exhibited potent tumor-suppressive effects and was more effective when administered vaginally than with the other two modalities.

Flagellar protein fibers are conserved structures used by bacteria for motility. A recent study where the self-assembly properties of flagella were used to prepare flagellar nanofibers (FNs) of different lengths reported that shorter FNs could activate higher antibody responses, indicating their potential to prepare cancer vaccines or adjuvants [149]. That study also reported the use of short FN as an adjuvant for the E6/E7 peptide of HPV-16 for intranasal administration in a mouse model of CC and found a substantial inhibition of tumors and prolonged survival in mice.

Heat shock protein 110 can act as a chaperone immune adjuvant to enhance the immune response to E749-57 of HPV-16 in mice [150]. Moreover, RGD (arginine–glycine–aspartate-containing peptide: ACRGDMFFCA) is a tumor-specific expression of the αvβ3 integrin-targeting peptide, which is widely used as a targeting ligand in studies on targeted cancer therapy [151]. For instance, the cationic peptide RGD-GGG-K18 was synthesized and wrapped in a negatively charged plasmid expressing E749-57-HSP110 to form a self-assembled nanoparticle tumor vaccine for the treatment of mice CC models by intraperitoneal injection [152]. The nanoparticles substantially inhibited tumor growth in mice, and this inhibition was produced by an immune response that depended on CD8+ T cells.

## 5. Discussion

An enhanced permeability and retention (EPR) effect exists in solid tumors, where particles sized between 10 and 200 nm are more readily taken up and retained for longer periods, and nanomedicines can be passively targeted [153,154,155]. However, this passive targeting of nanodrugs by relying only on the EPR effect has a major shortcoming: poor targetability [156]. Therefore, the development of nanomedicines with active targeting is a key focus for achieving precision medicine. Thanks to the various modifications associated with self-assembled peptide derivatives, their active targeting capabilities can be conferred or enhanced by design [157]. Some of CC’s biomarkers are common to multiple cancers, and therefore, some of the targeted drugs that are currently available for other cancers may also be utilized for the targeted treatment of CC. In addition to acting as drug delivery carriers, some self-assembled peptides or proteins show potential as oncology therapeutics in their own right. MeiYu Lv et al. developed the self-assembled peptide LMY1 to develop immunotherapeutic regimens for lung cancer [158]. LMY1 self-assembles into nanofibers after being cleaved by matrix metalloproteinase-2 (MMP-2) and has the ability to target cancers that highly express MMP-2, such as lung cancer and CC [159,160]. Tumor cells evade recognition and clearance by immune cells through CD47, whereas LMY1 self-assembled peptide fibrils can block the interaction between CD47 and signal-regulated protein α, thereby activating the phagocytosis of tumor cells by macrophages [161]. CD47 is also a target for CC therapy; thus, this self-assembled peptide may also have an inhibitory effect against CC [162]. Increasingly, more CC biomarkers are being discovered, and the targeted therapy technology for CC will be developed further [163].

In addition to being used to develop targeted therapeutics for BC and CC, self-assembled proteins and peptides can be used to assist in cancer-related research. For example, self-assembled peptide hydrogels (SAPHs) can be used to construct three-dimensional (3D) tumor models for cancer-related research. Three-dimensional tumor models can better simulate the complex structure of the extracellular matrix than the unnatural environment of a monolayer of cells in a two-dimensional (2D) matrix with uniform nutrients. In addition, such 3D models are more economical and time-efficient than animal models. Previous studies have used h9e peptide hydrogels to grow 3D cultures of BC cells, providing the cells with a 3D structure and allowing their easy collection for analysis by dilution followed by centrifugation [164].

Kun Mi et al. constructed 3D cultures of human BC cells using self-assembled peptide RADA16, matrix gel, and collagen I and found that BC cells exhibited different shapes in different matrices as well as lower proliferation and migration in RADA16 [165]. Subsequently, a xenograft model was constructed using a mixture of stroma and cells, revealing that stromal gel and collagen I promoted tumor growth in vivo. Meanwhile, the RADA16 group formed smaller tumors in vivo than the 2D cell control group, indicating that RADA16 had a growth-inhibitory effect on BC tumors. Another study showed that a subpopulation of (CD44+/CD24-) BC cells with gem/progenitor cell properties underwent substantial phenotypic reversal after 3D culture with RADA16 and that this phenotypic reversal prevented BC cells from forming tumors in nude mice [166].

Another study indicated that a beta-sheet-forming SAPH, PHPeptiGel®Alpha1, can be used to mimic BC progression in vitro or in vivo and is more applicable to the study of metastatic BC than to early-stage BC. The discovery of PeptiGel® Alpha1 uptake by BC cells suggests that PeptiGel® Alpha1 may have potential as a drug delivery vehicle [167]. Furthermore, PeptiGel®Alpha1 attenuated the inhibitory effect of tamoxifen (TAM) on BC cell viability, suggesting that it can reduce the cytotoxicity of some anticancer drugs—a property that may grant this SAPH some special applications.

Most BC patients undergo surgery to remove the tumor as a priority treatment; however, the inevitable loss of a portion of the breast due to tumor removal greatly affects the patient’s emotional health and may affect normal movement and other functions [168]. Therefore, breast reconstruction is also a key issue in the post-operative care of BC patients. Although autologous fat transplantation was the primary means of breast reconstruction in the past, autologous fat is absorbed slowly after transplantation and cannot survive for a long period [169]. Currently, there is evidence that SAPH can promote breast reconstruction. Huimin Wu et al. used a TAM-loaded RADA16-I peptide hydrogel to assist human adipose-derived mesenchymal stem cells (hADSCs) for breast reconstruction in nude mice [170]. TAM-RADA16-I provided an environment for hADSCs to attach and proliferate, while RADA16-I attenuated the toxic effect of TAM on hADSCs but maintained the toxic effect on BC cells, a difference that may be due to the fact that MCF-7 cells have more estrogen receptors. Thus, TAM-RADA16-I may have a role to play not only in breast reconstruction but also in preventing BC recurrence.

In conclusion, with the advancements in biomedical technology, nanoparticles are increasingly becoming attractive for use in targeted cancer therapy. Among them, self-assembled peptides and proteins have some notable advantages. The absorption of proteins and peptides clearly depends on the route of administration. Proteins are destroyed by gastric acid and digestive enzymes when administered orally and cannot be easily absorbed by the gastrointestinal tract; therefore, very few peptide drugs can be administered by this route. However, when used in mechanistic studies or for the development of targeted therapeutic agents, self-assembled peptides and proteins can play an important role in the treatment of BC and CC.

BC and CC are the two most common cancers in women worldwide, posing a tremendous threat to women’s health. Despite societal development in other domains, the morbidity and mortality rates of these two cancers have still not decreased, particularly among women who lack the awareness or conditions needed for prevention. Self-assembled proteins and peptides as delivery vehicles or drugs themselves can improve the shortcomings of existing therapeutic modalities, and continued advances in bioengineering may facilitate the design and production of functional nanomedicines, which hold great promise for the treatment and diagnosis of BC and CC. In particular, CC is one of the most common malignant tumors in women, posing a serious threat to women’s lives and health. Globally, 500,000 people die of CC every year, with most of the patients coming from low-income countries and regions. Traditional treatments for CC include surgery, radiotherapy, and chemotherapy. Although good results have been achieved with these treatments, there are some limitations and problems to be overcome. In recent years, with the emergence of new treatments such as immunotherapy and targeted therapy, the battle against CC has entered a new era.

## 6. Materials and Methods

Data sources and search strategy: in the PubMed database, “self-assembled peptides and proteins” OR “natural products” AND “breast cancer” OR “cervical cancer” OR “targeted cancer therapy” OR “drug delivery” were used as the subjects of the search, for which there was no time limit and the search remained open until 1 October 2023.

Literature selection criteria: the inclusion criteria were articles on natural products for the treatment and prevention of breast and cervical cancer and articles on self-assembled proteins and peptides for the treatment and prevention of breast and cervical cancer.

Exclusion criteria: non-natural products, non-self-assembled proteins and peptides, old or incomplete information, unclear description of the mechanism of action, unclear description of the test method, unclear description of the test indexes, inconsistency between the description of the test indexes and the results, conference papers, and the same experiment or the same batch of experiments.

Data extraction and processing: End Note x20 standard edition was used for literature management. The preliminary search returned 219 articles in PubMed and 333 articles in Web of Science, totaling 452 articles. After applying the criteria, 198 articles were excluded, leaving 254 articles. After reading the titles and abstracts, 99 documents were deleted because they did not match the subject matter, leaving 155 documents. After reading the full text and deleting 23 articles that did not meet the inclusion criteria, 122 articles were finally included, many of which only referred to the “approved resource for human gene nomenclature” for standardization of names, types, and other features of genes and proteins and their abbreviated descriptions. Excel 2019 was used to enter the information of article year, author, title, impact factor, signaling pathway, mechanism of action, and indicators of cervical and breast cancer treatment detection to establish the data file called “indicators of cervical and breast cancer treatment detection”.

## Figures and Tables

**Table 1:** Natural products and signaling pathways for the treatment of breast and cervical cancer.

Serial Number	Name	Cancer	Signaling Pathway	References
1	Rosemary extract	BC	AKT/mTOR	[81]
2	Astragalus polysaccharides	BC	PIK3CG/AKT/BCL2	[82]
3	Ethanol extract of Rhizoma Amorphophalli	BC	PI3K/AKT/mTOR	[83]
4	Sophoraflavanone G	BC	MAPK (AKT)	[84]
5	Rhus coriaria ethanolic extract	BC	ERK1/2, p38	[85]
6	Ganoderma lucidum extract	BC	IL-6/JAK2/STAT3 (JAK1/STAT3)	[86]
7	Extracts of Cordyceps sinensis	BC	NF-κB (macrophage)	[87]
8	Paeoniflorin	BC	Notch1	[88]
9	Nelumbo nucifera Gaertn. leaf extract	BC	PI3K/AKT/ERK (CTGF)	[89]
10	Ethanolic extract of Rhus coriaria	BC	NF-κB, STAT3	[90]
11	Aster tataricus, Vitex peduncularis Wall	CC	Caspase-3, -8, and -9, Bcl-2, -xL, survivin	[91]
12	Syzygium aromaticum	CC	Bax, PARP, caspase-3, ROS, Bcl-2, XIAP	[92]
13	Rhamnus sphaerosperma var. pubescens	CC	NO-, O2-, HOCl/OCl-, p-Akt	[93]
14	Root tubers and leaves of Ipomoea batatas	CC	CFP/YFP	[94]
15	Curcuma zedoaria	CC	p15, p53, Bax, cyclin D1, Bcl-2, MMP-2, -9, β-catenin, TCF7, c-Myc	[95]

**Table 2:** Drugs used clinically for breast cancer and their indications.

Serial Number	Name	Indications	Adverse Effects	Contraindications	References
1	Aromatase inhibitors	Adjuvant treatment of postmenopausal women with hormone receptor-positive early breast cancer.	Ulcers and blisters are common skin manifestations.	Hypersensitivity.	[96]
2	Raloxifene	Indicated for a risk reduction in invasive breast cancer in postmenopausal women, demonstrating a high risk for invasive breast cancer or women with osteoporosis.	Hot flashes, flu-like symptoms, muscle spasms, arthralgia, and infection.	Past medical history of deep venous thrombosis, renal vein thrombosis, pulmonary embolism, malignancy, active smoking, or any thrombophilia (factor V Leiden deficiency, prothrombin gene mutation G20210A, antiphospholipid syndrome, antithrombin deficiency, and protein c and s deficiency).	[97]
3	Cyclophosphamide	Use in the treatment of malignant lymphomas at stages III and IV.	Bladder and gonadal toxicity, hemorrhagic cystitis, amenorrhea, myelosuppression, alopecia, and spells of nausea and vomiting.	Patients with allergies or hypersensitivity reactions to the drug or any of its metabolites.	[98]
4	Pamidronate	Moderate or severe hypercalcemia of malignancy, moderate-to-severe Paget’s disease of bone, osteolytic bone metastases of breast cancer, and osteolytic lesions of multiple myeloma	Hypocalcemia and resulting secondary hyperparathyroidism, acute phase response, musculoskeletal pain, various ocular events, and osteonecrosis of the jaw.	Those with hypersensitivity to bisphosphonates or mannitol.	[99]
5	Trastuzumab	HER2-positive breast cancer: adjuvant therapy.	Pregnancy disruption and cardiac dysfunction.	Cardiotoxicity, usually manifested as a decrease in the left ventricular ejection fraction (LVEF).	[100]
6	Tamoxifen	Treatment of breast cancer in both females and males.	Uterine malignancies, pulmonary embolism, and stroke in patients who are at high risk for cancer or who have ductal carcinoma in situ.	The drug or any component in its formulation or concomitantly with warfarin.	[101]
7	Fulvestrant	HR-positive and HER2-negative breast cancer.	Pain at the injection site and hot flashes.	Women who are pregnant or breastfeeding.	[102]
8	Doxorubicin	For acute leukemia (lymphoblastic and granulocytic), malignant lymphoma, breast cancer, lung cancer (small cell and non-small cell lung cancer), ovarian cancer, bone and soft tissue sarcoma, nephroblastoma, bladder cancer, thyroid cancer, prostate cancer, squamous carcinoma of the head and neck, testicular cancer, gastric cancer, and liver cancer.	Bone marrow suppression, cardiotoxicity, gastrointestinal reactions: manifested as loss of appetite, nausea, vomiting, but also oral mucosal erythema, ulcers and esophagitis, gastritis, and alopecia. Fever, hemorrhagic erythema, liver function abnormalities, etc.	Gastrointestinal obstruction, jaundice, or hepatic impairment patients; patients with cardiopulmonary failure, chickenpox; or herpes zoster; patients who have been treated with other antitumor drugs or radiation therapy that has caused bone marrow suppression is contraindicated; patients with severe heart disease are contraindicated. contraindicated in pregnant and lactating women.	[104,105]
9	Docetaxel	Good efficacy in advanced breast cancer, ovarian cancer, and non-small cell lung cancer.	Bone marrow suppression: dose-limiting toxicity is neutropenia; allergic reactions: mild allergic reactions manifested as itching, flushing, rash, drug fever, chills, etc., severe allergic reactions are rare, characterized by bronchospasm, dyspnea, and hypotension; skin reactions: mainly in the hands and feet, but also in the arms, face and chest rash, which may be accompanied by itching; nausea, vomiting, and diarrhea, alopecia, malaise, mucositis, arthralgia, myalgia, injection site reactions, neurotoxicity and cardiovascular toxicity.	Contraindicated in persons who are hypersensitive to the product. It is contraindicated in persons with severe bone marrow suppression, severe hepatic, or renal impairment, and in pregnant and lactating women.	[106,107]
10	Methotrexate	It is indicated for systemic use in the treatment of choriocarcinoma of the epithelium, all types of acute leukemia, breast cancer, lung cancer, head and neck cancer, and cervical cancer. High-dose methotrexate supplemented with calcium formyltetrahydrofolate rescue (HDMTX-CFR therapy) has certain efficacy as postoperative adjuvant chemotherapy or systemic therapy for advanced lesions of malignant lymphoma, acute lymphoblastic leukemia, breast cancer, ovarian cancer, small-cell lung cancer, and so on.	Gastrointestinal reactions are mainly stomatitis, mouth and lip ulcers, pharyngitis, nausea, vomiting, gastritis and diarrhea. Bone marrow suppression is mainly manifested as a decline in white blood cells, platelets also have a certain effect, in severe cases, whole blood decline, skin or visceral bleeding can occur. A large number of applications can lead to serum alanine aminotransferase (ALT) elevation, or drug hepatitis, a small amount of persistent application can lead to hepatic cirrhosis.	Causes kidney damage at high doses and can be teratogenic when used early in pregnancy.	[110,111]
11	Fluorouracil	It is used alone or in combination with other agents in the adjuvant treatment of breast cancer surgery and in the palliative treatment of some non-surgical malignancies, particularly those of the breast and pancreas. The combination of cyclophosphamide and MTX (breast cancer) often results in high response rates and survival rates.	Bone marrow suppression: mainly leukopenia, platelet drop. Loss of appetite, nausea, vomiting, stomatitis, gastritis, abdominal pain and diarrhea and other gastrointestinal reactions; injection of local pain, phlebitis or arterial endarteritis, often alopecia, erythematous dermatitis, skin pigmentation hand-foot syndrome and temporary cerebellar motor disorders, and occasionally affects the function of the heart.	Blood test should be strictly checked during the use of the drug. Keep in a dark place away from light, the temperature should not be lower than 10 °C, and it should not exceed 35 °C. Inflammation in the area of application during the treatment period, the inflammation will subside after stopping the drug. This product can cause severe skin irritation, especially in the sun.	[109,113].
12	Orserdu(elacestrant)	Treatment of postmenopausal women or adult men with advanced or metastatic ER-positive, HER2-negative, and ESR1-mutated breast cancer that has progressed after at least one endocrine therapy.	Musculoskeletal pain, nausea, elevated cholesterol, increased AST, increased triglycerides, fatigue, decreased hemoglobin, vomiting, increased ALT, decreased sodium, increased creatinine, decreased appetite, diarrhea, headache, constipation, abdominal pain, hot flashes, and indigestion.	Lactation: breastfeeding is not recommended. Hepatic impairment: avoid use in patients with severe hepatic impairment (Child–Pugh C). Reduce dose in patients with moderate hepatic impairment (Child–Pugh B).	[114]
13	Truqap(capivasertib)	In combination with fulvestrant for the treatment of adult patients with hormone receptor (HR)-positive, human epidermal growth factor receptor 2 (HER2)-negative, locally advanced, or metastatic breast cancer with one or more PIK3CA/AKT1/PTEN mutation as detected by an FDA-approved trial, who have progressed after treatment with at least one endocrine-based regimen in metastatic disease, or who have relapsed within 12 months of completing adjuvant therapy.	Musculoskeletal pain, nausea, elevated cholesterol, increased AST, increased triglycerides, fatigue, decreased hemoglobin, vomiting, increased ALT, decreased sodium, increased creatinine, decreased appetite, diarrhea, headache, constipation, abdominal pain, hot flashes, and dyspepsia.	Dyslipidemia: may result in hypercholesterolemia and hypertriglyceridemia. Monitor lipids before and periodically after initiating therapy. Embryo-fetal toxicity: can lead to fetal harm. Advise of potential risks to the fetus and use effective contraception.	[117,118]
14	Verzenio(abemaciclib)	Patients with advanced or metastatic HR+ and HER-2- breast cancer; in patients whose disease has worsened after hormonal therapy for breast cancer, it may be combined with flugestone; patients with early-stage breast cancer who are receiving chemotherapy for distant metastases; and the combination of abciximil and an aromatase inhibitor may be used as a first-line hormonal treatment option for breast cancer in postmenopausal women.	Diarrhea, neutropenia, nausea, abdominal pain, infection, fatigue, anemia, neutropenia, decreased appetite, vomiting, headache, and thrombocytopenia	Avoid concomitant use of ketoconazole. Decrease concomitant use of VERZENIO administration with other strong CYP3A inhibitors; avoid concomitant use of strong CYP3A inducers; advise against breastfeeding during lactation.	[123]

## Data Availability

No new data were created or analyzed in this study. Data sharing is not applicable to this article.
